# Five Underutilized Ecuadorian Fruits and Their Bioactive Potential as Functional Foods and in Metabolic Syndrome: A Review

**DOI:** 10.3390/molecules29122904

**Published:** 2024-06-19

**Authors:** Rodrigo Duarte-Casar, Nancy González-Jaramillo, Natalia Bailon-Moscoso, Marlene Rojas-Le-Fort, Juan Carlos Romero-Benavides

**Affiliations:** 1 Tecnología Superior en Gestión Culinaria, Pontificia Universidad Católica del Ecuador Sede Manabí, Portoviejo 130103, Ecuador; rduarte@pucesm.edu.ec (R.D.-C.); erojas@pucem.edu.ec (M.R.-L.-F.); 2 Maestría en Alimentos, Facultad de Ciencias Exactas y Naturales, Universidad Técnica Particular de Loja, Loja 110108, Ecuador; negonzalez4@utpl.edu.ec; 3 Facultad de Ciencias de la Salud, Universidad Técnica Particular de Loja, Loja 110108, Ecuador; ncbailon@utpl.edu.ec; 4 Departamento de Química, Facultad de Ciencias Exactas y Naturales, Universidad Técnica Particular de Loja, Loja 110108, Ecuador

**Keywords:** tropical fruits, Ecuadorian Amazon, underutilized fruits, bioactive potential, phenolic compounds, carotenoids, traditional medicine, sustainable agriculture, functional foods, nutraceuticals

## Abstract

The Ecuadorian Amazon harbors numerous wild and cultivated species used as food, many of which are underutilized. This review explores the bioactive potential of five such fruits—Borojó (*Alibertia patinoi*); Chonta (*Bactris gasipaes*); Arazá (*Eugenia stipitata*); Amazon grape (*Pourouma cecropiifolia*), a wild edible plant; and Cocona (*Solanum sessiliflorum*)—and their applications against metabolic syndrome. This study highlights their health-promoting ingredients and validates traditional medicinal properties, emphasizing their significance in improving health and mitigating the effects of the Western diet. These fruits, integral to Ecuadorian cuisine, are consumed fresh and processed. Chonta is widely cultivated but less prominent than in pre-Hispanic times, Borojó is known for its aphrodisiac properties, Cocona is traditional in northern provinces, Arazá is economically significant in food products, and Amazon grape is the least utilized and researched. The fruits are rich in phenolics (*A. patinoi*, *E. stipitata*) and carotenoids (*B. gasipaes*, *E. stipitata*), which are beneficial in controlling metabolic syndrome. This study advocates for more research and product development, especially for lesser-known species with high phenolic and anthocyanin content. This research underscores the economic, cultural, and nutritional value of these fruits, promoting their integration into modern diets and contributing to sustainable agriculture, cultural preservation, and public health through functional foods and nutraceuticals.

## 1. Introduction

Food does not only feed and nourish, it is also an agent of either health or illness. The study of the health-promoting qualities of foods has begotten the field of nutraceuticals, defined as “food or part of food that offers medical/health benefits including the prevention and treatment of diseases” [1]. One of the health-promoting properties of nutraceuticals is their ability to help prevent and undo the damage that the Western pattern diet (WPD) causes in the form of metabolic syndrome (MetS), a public health burden and a global epidemic [2] compounded by an aging population and unhealthy lifestyles [3].

The increase in noncommunicable diseases since the beginning of industrial development, which led to a change in lifestyle, allows us to see the impact of food on our health, so this review seeks to introspect on the use of available natural resources and the benefits of consuming traditional fruits, as well as scientifically validating the medicinal properties that have been attributed to them since ancient times and promoting the use of by-products derived from the processing of these fruits for their phytochemical qualities and possible uses as food and supplements.

Obesity has become a worldwide public health problem, initiating at increasingly earlier ages [4]; there are three critical stages wherein greater control must be exerted to prevent its development: prenatal, from five to seven years old where the increase in adipose tissue is evident, and adolescence [5,6]. Being a multifactorial disease, it is related to physical, systemic, and physiological processes, resulting in disorders associated with chronic diseases such as type 2 diabetes, hypertension, and dyslipidemia, which increases the risk of cardiovascular accidents and is known as metabolic syndrome [7,8,9]; in addition, it is also related to fatty liver and musculoskeletal disorders, as well as sleep apnea, alterations in cognitive function, and psychological disorders such as depression, which makes it more likely that the mortality rate during early adulthood will increase, ranging from 21 to 40 years old [10,11].

There are several conditions that play a role against healthy diets and which allow for the development of obesity, starting with economic globalization with which a food transition was implemented consisting of diets rich in animal products, ultra-processed foods with high fat content and, mainly [12], the production of an imbalance between caloric consumption and its expenditure, due to sedentary life and its consumption. In addition to this, particularly in children, they are indiscriminately exposed to advertisements broadcast by various media that promote a consumer culture in terms of food and beverages with low nutritional content, as well as ultra-processed and unhealthy due to the number of additives included in the food formulas [13,14].

Another factor that amplifies the problem is self-medication, referring to the consumption of antibiotics that enhance the probability of death from infections caused by multi-resistant bacteria and which, due to the fact that they are not selective, produce dysbiosis [15]. A diet high in fat and low in fiber promotes the growth of Gram-negative pathogens within the intestine and makes the individual more prone to intestinal inflammatory processes in the future [16]; the incorporation of treatments with antibiotic substitutes according to the case or adjuvants can reduce the problem; however, dietary intervention can help restore the gut microbiota that are responsible for multiple regulatory functions such as metabolism, neuronal modulation, and development of the immune system [14,15].

A balanced daily diet at all stages of life provides our body with the necessary means to stay healthy [12]. For this reason, the aim is to boost the consumption of antioxidants through food, since after being absorbed in the small intestine or bio-transformed by the gut microbiota, they modulate various mechanisms of action that are related to the prevention of diseases; however, it is important to mention that the benefits depend on the concentration ingested since their excessive consumption can be counterproductive by acting as pro-oxidants and generating toxicity [8,13,14].

Plants and other organisms synthesize secondary metabolites to perform various functions for them such as signaling, insect and ruminant repellant, antimicrobial, and prevention of oxidative damage; they also impart organoleptic characteristics that include color, flavor, and aroma [17]. Several of these secondary metabolites exhibit antioxidant activity.

Tropical America is home to a very wide variety of plant species, many of which remain poorly investigated. Rediscovered ancestral staples neglected following the European conquest, such as *Chenopodium quinoa*, *Amaranthus* spp., and *Bactris gasipaes,* have garnered great interest for their food security and functional potential [18,19]. Other species of interest have traditionally been sources of food and medicine, but have not attained staple status, and also provide important nutrients and phytochemicals. Examples among these species that are being made known in global markets are *Alibertia patinoi*, regarded as an aphrodisiac and energizer; *Ilex guayusa,* of which the energizing properties have made it into popular drink products; and *Euterpe oleracea,* which has attained the dubious “superfood” status [20,21,22]. This status is a marketing and consumer culture term, not a science one. It refers to species high in bioactive compounds but is often misleading, opening the possibility of food fraud and unsubstantiated claims [23,24]. The popularization of exotic products is not per se a good thing, as it may impact the livelihood of those that traditionally consume it, as is the case with *E. oleracea* and *C. quinoa*, and it must therefore be monitored to ensure that food security is not threatened [25,26].

Fruits that grow in Ecuador have largely been understudied, and there is great potential in their variety and abundance [27]. Tropical fruits represent around 10% of the world’s fruit market [28], and Ecuador, located entirely in the intertropical region, is rich in traditional and exotic fruits. Among these, lesser-known Amazonian fruits hold great phytochemical and bioactive potential.

This work aims to summarize the current knowledge about five underutilized—or minor—species through a literature review. The species are the following: Arazá (*E. stipitata*), Cocona (*S. sessiliflorum*), Chonta (*B. gasipaes*), Borojó (*A. patinoi*), and Amazon grape (*P. cecropiifolia*). This works seeks to validate the medicinal properties that are attributed to them and explore the potential health benefits of the consumption of these species, as well as products and byproducts derived from them, for both their functional and pharmacological properties, highlighting promising compounds found in them and envisioning future research lines to explore the potential of the species, with emphasis on their applications relative to obesity and metabolic syndrome.

These fruits present different levels of underutilization. They are all native to the Ecuadorian Amazon and cultivated by small farmers and small-to-medium businesses, except for *B. gasipaes*, a main species for the sustainable production of hearts of palm. However, as a fruit, it is not an important product [19]. *E. stipitata* is popular in the Amazon region and is cultivated and selected for industrial production [29]. *A. patinoi* is being marketed as a “superfood” with energizing and aphrodisiac properties [20]. *S. sessiliflorum* is appreciated as fruit and medicine, both as a wild and as a cultivated species, with initiatives toward larger-scale production [30,31]. *P. cecropiifolia* is largely not cultivated, but it is harvested as a wild edible plant (WEP) [30,32].

The summary of our findings is as follows: the most studied fruits are those that are most cultivated, and the WEP is the least studied among the selected fruits. The studied fruits are rich in phytochemicals, namely carotenoids and phenolics, and *P. cecropiifolia* are rich in anthocyanins, which are all bioactive molecules with activity against MetS, in line with the traditional uses of the species. *A. patinoi* has the most patents, followed by *B. gasipaes*, several of which address the proven or purported biological activity of the species. The studies on the species mainly address their food uses, presenting an opportunity for deepening the knowledge of their functional and pharmacological uses.

### Context

Fruits and vegetables are an abundant source of antioxidants, such as polyphenols, carotenoids, and vitamins (Table 1), suitable for human consumption [15,33], and depending on the amount ingested and their bioavailability given by their chemical structure and interaction with the physiological conditions of the consumer, it is possible to achieve an equilibrium between the formation and neutralization of prooxidants that is strongly related to the development of non-communicable diseases. Therefore, they play a vital role in the human diet and attempts are made to prevent their loss during processing and storage [14,34,35].

Tropical fruit is a growing segment of the fruit industry, both as fresh fruit and as food products. The most traded tropical fruits worldwide are banana (*Musa paradisiaca*), pineapple (*Ananas comosus*), mango (*Mangifera indica*), avocado (*Persea americana*), and papaya (*Carica papaya*) [38].

## 2. Method

The document analysis was performed through the Scientific Procedures and Rationales for Systematic Literature Reviews (SPAR-4-SLR) protocol [39]. The search was performed in scientific databases (Scopus, Crossref) using title, abstract, and keywords, under the taxonomic names of the species, both current and synonyms; for example, in the case of *A. patinoi*, both “*Alibertia patinoi*” and “*Borojoa patinoi*” were included in the search terms. The time span was between 2011 and 2024. The selected documents were articles, reviews, and book chapters in English, Spanish, or Portuguese. Preprints, proceedings, notes, and errata were excluded from the document search. The results were deduplicated and the content of the documents was assessed by reading the abstracts and the documents, including those that dealt with MetS, food, nutrition, and phytochemistry. The final dataset comprised 110 documents, and the procedure is summarized in Table 2.

From the results, research categories according to Australian and New Zealand Standard Research Classification (NZSRC) and Sustainable Development Goals (SDGs) were obtained from Dimensions [44,45].

Research trends analysis was performed in Bibliometrix/biblioshiny 4.0 and VOSviewer 1.6.20 [46,47].

## 3. Overview

**Figure 1:**
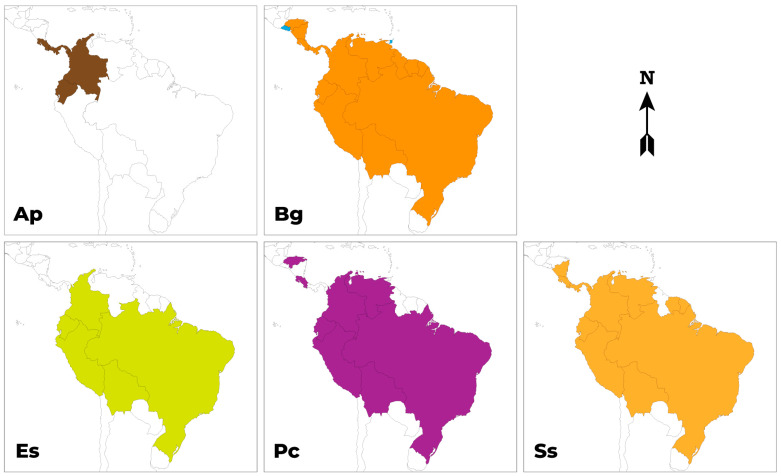
Geographic distribution of the selected species, by country. Ap, *A. patinoi*. Bg, *B. gasipaes*. Es, *E. stipitata*. Pc, *P. cecropiifolia*. Ss, *S. sessiliflorum*.

The studied species are native to tropical America, mostly South America and the Amazon basin, and also countries in Central America (Figure 1). Only *B. gasipaes* is considered an introduced species in El Salvador and Trinidad-Tobago [48].

A scheme of the research volume, categories, and Sustainable Development Goals (SDGs) for the studied fruits is shown in Table 3.

*B. gasipaes* shows the highest research volume, consistent with its traditional, historical, and current economic importance. *E. stipitata*, also of industrial importance, is second among the selected species. *S. sessiliflorum*, *A. patinoi*, and *P. cecropiifolia* are progressively less studied: this is in line with their relative economic importance. None of the studied fruits are important tropical fruits by volume. Reviews mention *E. stipitata* and *B. gasipaes* as frequently cultivated Amazon fruits at the domestic level [41,50]. A study in species use frequency in indigenous communities in the Colombian Amazon lists the species in this study in descending abundance: *B. gasipaes* (786), *A. patinoi* (700), *P. cecropiifolia* (393), *E. stipitata* (292), and *S. sessiliflorum* (250) [51].

Research categories are consistent among the five species: Agricultural, Veterinary, and Food Sciences; Biological Sciences; and Food Science are the predominant categories, which implies that the main interest in the studied species is as food, with Biomedical and Clinical Sciences and Engineering taking a prominent, but less principal place as research subjects.

Sustainable Development Goals (SDGs) are still underrepresented in the research, with SDG 2: Zero Hunger; SDG 3: Good Health and Well-Being; SDG 7: Affordable and Clean Energy; SDG 12: Responsible Consumption and Production; SDG 13: Climate Action; SDG 14: Life Below Water; and SDG 15: Life on Land listed, with SDG 15 present in all species except for *S. sessiliflorum*. Zero hunger, which would be the main SDG when studying food species, appears for three of the five species.

### 3.1. Alibertia patinoi

**Figure 2:**
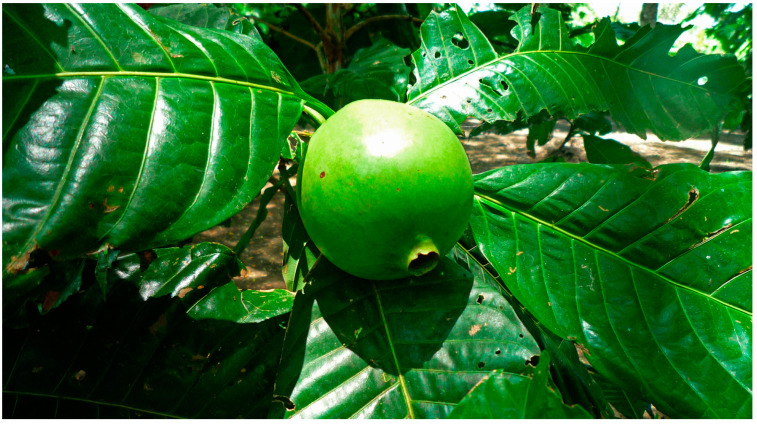
*Alibertia patinoi* unripe fruit. Image by Jean-Luc Crucifix, CC BY 3.0.

*Borojó* is a traditionally appreciated fruit that grows in the Pacific coast of Colombia and Ecuador and in the Amazon. *Alibertia patinoi* is a plant species in the *Rubiaceae* family that is native to Colombia, Ecuador, and Peru. Its vernacular name means “tree of the hanging heads” due to the similarity of the size and shape of the fruit with a human head (Figure 2) [52]. 

Ethnopharmacological uses of *A. patinoi* related to MetS include blood pressure control, antimicrobial, wound-healing, and anticancer [20,53].

### 3.2. Bactris gasipaes

**Figure 3:**
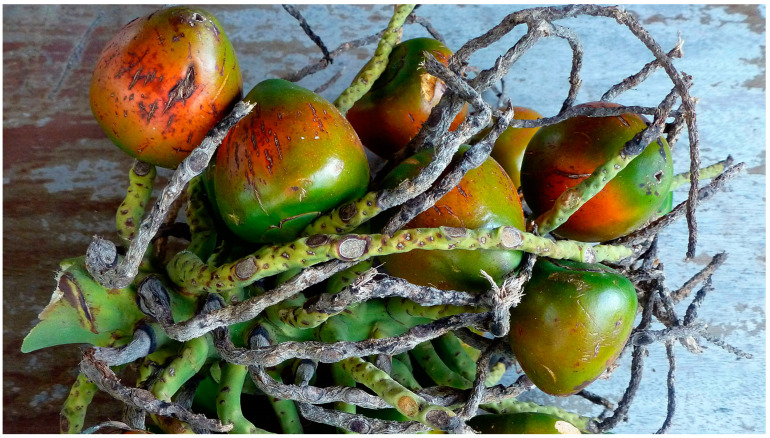
*Bactris gasipaes* fruit cluster. Image by Kalamazad Khan, CC BY-SA 4.0.

Peach palm is a staple of the Amazon people, domesticated around 4000 years ago and which, although neglected, has made a comeback as a crop. It is a fruit-bearing palm tree native to the tropical regions of South and Central America. The fruit and seeds of this plant are traditionally consumed in these regions and are also gaining popularity in other parts of the world. There are two main varieties: *gasipaes*, cultivated and bearing larger fruit (Figure 3), and *chichagui*, wild and bearing smaller, oilier fruit [19].

Ethnopharmacological uses of *B. gasipaes* related to MetS include anti-inflammatory, antimicrobial, and anticancer [30,54]. 

### 3.3. Eugenia stipitata

*E. stipitata*, also known as “Arazá” or “Araça Boi”, is a fruit-bearing tree native to South America. The fruit of this plant is highly valued for its unique and sour flavor and aroma. There may be confusion when searching for the species using its vernacular name because the name *arazá* is also used for other species, such as *Psidium* spp. [55].

**Figure 4:**
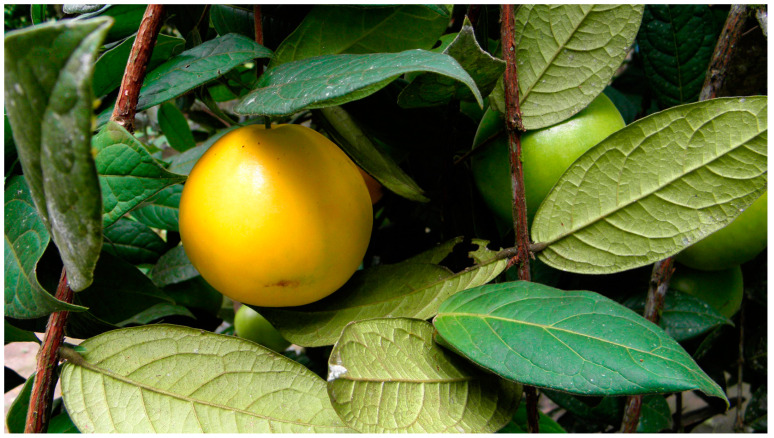
*Eugenia stipitata* ripe fruit, unripe fruit, and leaves. Image by Luis Alveart, CC BY-NC-ND 2.0.

It appears to have been domesticated and disseminated from its origin in Peru by the Eastern Tucanos, a people that live in what today is the border between Colombia and Brazil. Of the two subspecies *stipitata* and *sororia*, the latter (Figure 4) is the most suitable for cultivation [56]. It is a delicate, susceptible fruit that requires great care in cultivation and postharvest handling [57]. Species from the *Eugenia* genus, including *E. stipitata*, show a promising phytochemical profile against diabetes [58], and *E. stipitata* has been traditionally used in the treatment of several ailments, mainly bladder and intestinal problems [57]. 

Ethnopharmacological uses of *E. stipitata* related to MetS center on its antioxidant and antimicrobial activity [59,60]. 

### 3.4. Pourouma cecropiifolia

**Figure 5:**
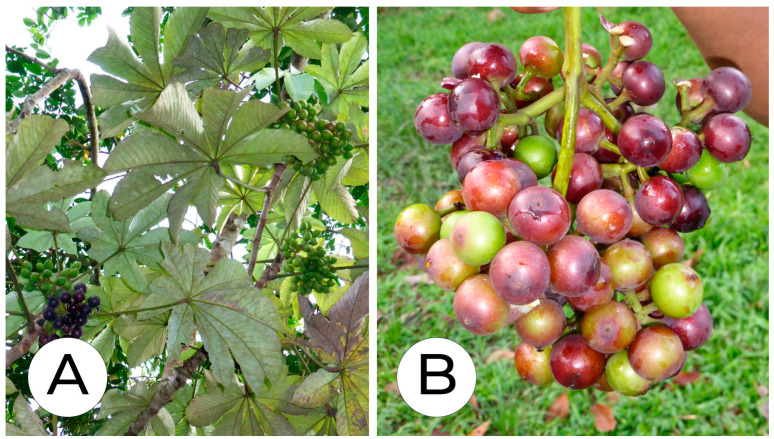
*Pourouma cecropiifolia*. (**A**): Tree and unripe and ripe fruit. (**B**): Fruit. Images by Kristof Zyskowski and Yulia Bereshpolova, CC BY 2.0.

*P. cecropiifolia* is a plant species native to the Amazon rainforest of South America. It is a member of the *Moraceae* family and is also known as “almendro”, “biriba”, or “uva caimarona” (Figure 5). It contains several phytochemicals with potential health benefits. It has been compared with acai (*Euterpe oleracea*) due to its antioxidant activity, but it is barely cultivated in domestic plots due to the height at which the fruit is borne in the tree, and remains a WEP [32,61]. The species appears to have no reported ethnomedical uses [30].

### 3.5. Solanum sessiliflorum

**Figure 6:**
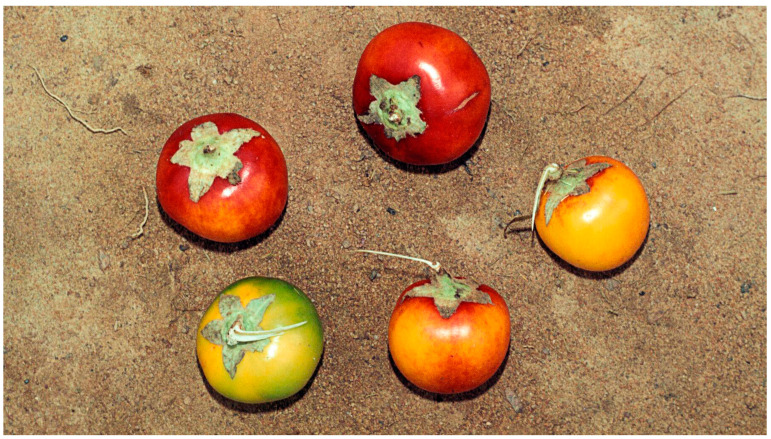
*Solanum sessiliflorum*. Image by Marie-Françoise Prévost CC-BY-SA.

*S. sessiliflorum*, locally known as “cocona” (Figure 6), is a fruit-bearing plant native to the Amazon rainforest of South America. It is a member of the *Solanaceae* family and the Solanum genus, abundant in bioactive phytochemicals [62]. It is traditionally used as a treatment for diabetes and in wound-healing [63].

### 3.6. Nutritional properties

Fruit is naturally sweet, and thus tends to be high in carbohydrates. The main nutritional properties of the studied fruits are summarized in Table 4. *Alibertia patinoi* is traditionally part of the food security in the norther Ecuadorian and southern Colombian Pacific region, as well as among the Amazon communities [51]. The fruit is high in energy, minerals, and particularly calcium, phosphorus, and iron. The pulp is naturally acidic (pH 3.5) [20]. *Bactris gasipaes* is also a species that represents food security in tropical America [64,65]. It a good source of carbohydrates, vitamins A and E, and heart-healthy fats, such as 36% oleic and 11–21% linolenic acids [19,66,67]. The white variety is richer in minerals than yellow or red varieties [68]. *Eugenia stipitata* is a nutritious fruit with double the vitamin C content of oranges [57], rich in magnesium and copper, although most phytochemicals reside in the seeds [69]. *Pourouma cecropiifolia* is rich in carbohydrates and a source of vitamins B3 and C. *Solanum sessiliflorum* is a good source of potassium and vitamin C.

Nutrient-wise, the studied fruits are sources of energy, micronutrients, and the seeds can be sources of oils with a healthy lipidic profile, due to the presence of mono and polyunsaturated fatty acids such as oleic, linoleic, palmitoleic, and others.

## 4. Biological Activity

Besides the antioxidant capacity of a wide variety of secondary metabolites present in fruit, there is an array of biological activity in the selected fruits, presented in Table 5. The most active fruits are *A. patinoi* and *E. stipitata*.

*A. patinoi* exhibits antimicrobial, antitumor, cytotoxic, spermicide, and skin protective activity. *B. gasipaes* presents anti-inflammatory, antimicrobial, and hepatoprotective activity. *E. stipitata* does not exhibit cytostatic effect, but it has antigenotoxic and antimutagenic activity [80]. Its anthocyanin-rich extracts show activity against larynx, liver, and breast cancer cell lines, while the pure compounds do not [81]. Its hydroalcoholic extracts inhibit acetylcholinesterase, of interest for the improvement of the symptoms of Alzheimer’s disease [82]. This species is the least studied, and its anthocyanin-rich extracts exhibit promising activity. *S. sessiliflorum* shows antimicrobial, cytotoxic, hypoglucemiant, cytoprotective, and antiproliferative effects. It exhibits in vitro biological activity against lipid peroxidation, which is of interest concerning MetS [83].

**Table 5:** Biological activity of extracts and powders of the studied fruits.

Activity	Ap	Bg	Es	Pc	Ss	Ref.
Antigenotoxic			X		X	[84]
Anti-inflammatory		X	X			[63,85]
Antimicrobial	X	X	X		X	[53,83,86,87]
Antimutagenic			X			[80]
Antitumor	X					[53]
Antitumor activity regulation	X					[53]
Cytoprotective					X	[83]
Cytotoxic	X		X	X	X	[27,84]
Hepatoprotective		X				[88]
Hypoglucemiant		X			X	[69,88]
Skin protection	X		X			[89]
Spermicide	X					[90]

Ap, *A. patinoi*. Bg, *B. gasipaes*. Es, *E. stipitata*. Pc, *P. cecropiifolia*. Ss, *S. sessiliflorum*.

### 4.1. Anti-Inflammatory

*B. gasipaes* carotenoid-rich extracts exhibit nephroprotective effect through antioxidant and anti-inflammatory action [85,86]. Ethanolic extracts of *S. sessiliflorum* exhibit anti-inflammatory and wound-healing effects in animal models [63].

### 4.2. Anticancer

The aqueous extract of the peel and pulp of *A. patinoi* exhibits cytotoxic activity against WKD and Caco-2 colon cancer cell lines, and iridoids from its extracts exhibit antiproliferative effect [53,91].

The ethanolic extract of the pulp of *E. stipitata* shows antigenotoxic and antimutagenic activity, presumably due to its antioxidant capacity [80].

Anthocyanin-rich extracts from *P. cecropiifolia* using methanol: acetic acid exhibits “promising cytotoxic effects on larynx, gastric, and breast cancer cell lines” [81].

### 4.3. Antimicrobial

The aqueous extract of the peel and pulp of *A. patinoi* possesses promising antimicrobial activity against multi-resistant strains of *Pseudomonas aeruginosa*, *Staphylococcus aureus*, and *Candida* species [53].

### 4.4. Hypoglucemiant and Hypolipemiant

*B. gasipaes* and *S. sessiliflorum* exhibit hypoglucemiant activity attributable to their carotenoid content, and also fat reduction in pork meat [19,69,88,92].

### 4.5. Other Activity

*A. patinoi* extracts have spermicidal effect [90]. Acetone extract of *B. gasipaes* pulp has hepatoprotective effect against oxidative stress in rats (IC_50_ = 10.9 μg/mL) [88].

The ethanolic extract of *E. stipitata* seeds possesses significant anthelmintic activity against ovine gastrointestinal nematodes [93].

Antimicrobial activity and cytotoxicity are recurrent biological activities of the species. *P. cecropiifolia* shows little biological activity that can perhaps be attributed to the dearth of research on the species.

## 5. Phytochemical Composition and Activity

The studied fruits contain a variety of phytochemicals, with different compositions per species.

*A. patinoi* contains a variety of phytochemicals with potential health benefits. Among these, the most salient are flavonoids, such as catechin, quercetin, and kaempferol, which have antioxidant properties and can help protect cells from oxidative stress. They also have anti-inflammatory, antiviral, and anticancer activities [94]. Other phenolic compounds, such as chlorogenic, caffeic, and other hydroxycinnamic acids, also have antioxidant properties and can help prevent the development of chronic diseases, such as cancer and cardiovascular diseases [95]. *A. patinoi* also contains oleuropein and phloridzin, which exhibit several beneficial effects, presumably due to their antioxidant activity. These phytochemicals could partly explain the aphrodisiac fame the fruit has [20].

*B. gasipaes* contains carotenoids, mainly beta-carotene, lycopene, and zeaxanthin. Carotenoids are known for their antioxidant properties and can help protect cells from oxidative stress. They also have anti-inflammatory and immunomodulatory activities [96]. Its lipid profile is rich in unsaturated fatty acids such as oleic and linoleic, which are components of heart-healthy fats and oils [19]. Palmitic acid is the main fatty acid in the species, which on its own is not considered heart-healthy, but the oil as a whole has been regarded as heart-healthy and found to increase HDL cholesterol and lower BMI in animal models [66,67].

*E. stipitata* contains several phytochemicals with potential health benefits [97]. Among them are polyphenols, including hydroxycinnamic acids and flavonoids, particularly myricetin at 17 mg/100 g fresh pulp [69,80]. These compounds have antioxidant properties and can help protect cells from oxidative stress. They also have anti-inflammatory and anticancer activities. *E. stipitata* also contains carotenoids, including beta-carotene, zeaxanthin, and a characteristically high proportion of lutein [98]. These compounds have recognized antioxidant properties and can help protect cells from oxidative stress. They also have anti-inflammatory and immunomodulatory activities [98]. *E. stipitata* is a good source of vitamin C, an important antioxidant that can help protect cells from oxidative stress. Vitamin C also has anti-inflammatory and immunomodulatory activities [99]. *E. stipitata* seeds are a source of fatty acids, including oleic and linoleic acids. These compounds have several health benefits, including cardiovascular and anti-inflammatory effects.

*P. cecropiifolia* is rich in flavonoids, mainly rutin, and also quercetin, kaempferol, and their glycosides. Flavonoids are known for their antioxidant properties and can help protect cells from oxidative stress. They also have anti-inflammatory, antiviral, and anticancer activities [94,100]. The species also contains anthocyanins, mainly cyanidin-3-glucoside (244.57 ± 2.13 mg/kg fresh fruit) and delphinidin-3-glucoside (104.42 ± 2.45 mg/kg fresh fruit) [101]. These compounds have antioxidant properties and can help protect cells from oxidative stress [102], and they are present in a concentration similar to that of blackcurrants [103].

*S. sessiliflorum* is rich in carotenoids: beta-carotene and all-(E) lutein. Carotenoids are known for their antioxidant properties and can help protect cells from oxidative stress. They also have anti-inflammatory and immunomodulatory activities. The main phenolic in the species is 5-caffeoylquinic acid, which makes its extracts powerful antioxidant scavengers [104]. There is qualitative indication of alkaloid presence, but no positive identification [105]. Among the compounds of interest in *S. sessiliflorum* are caffeic acid, the derivatives of which have been patented as hypoglucemiants [106]. Chlorogenic acid is used in the control of diabetes type 2 in the form of, among others, Emulin™, a patented blend of chlorogenic acid, myricetin, and quercetin [107], which is also sold as a dietary supplement. Rutin is cardioprotective, but due to its low bioavailability, it has not found its way into drugs [108].

**Figure 7:**
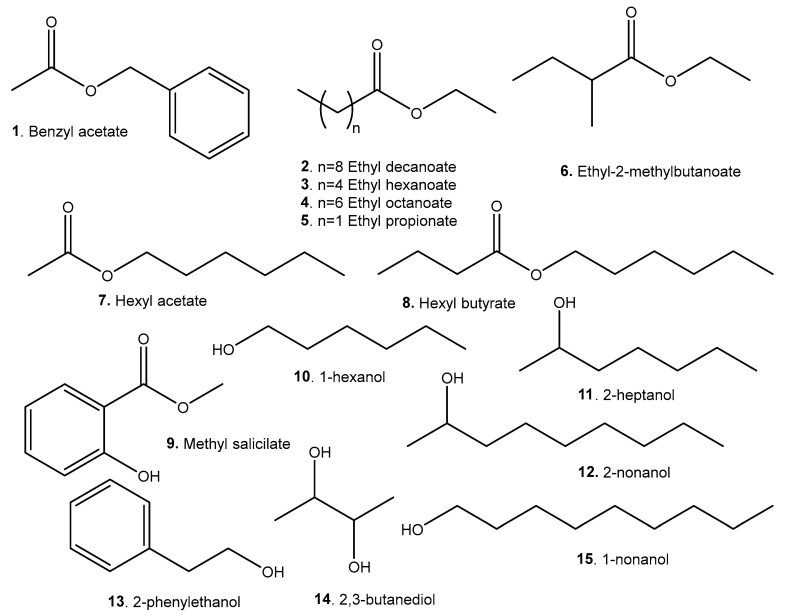
Esters and alcohols present in the studied species.

**Figure 8:**
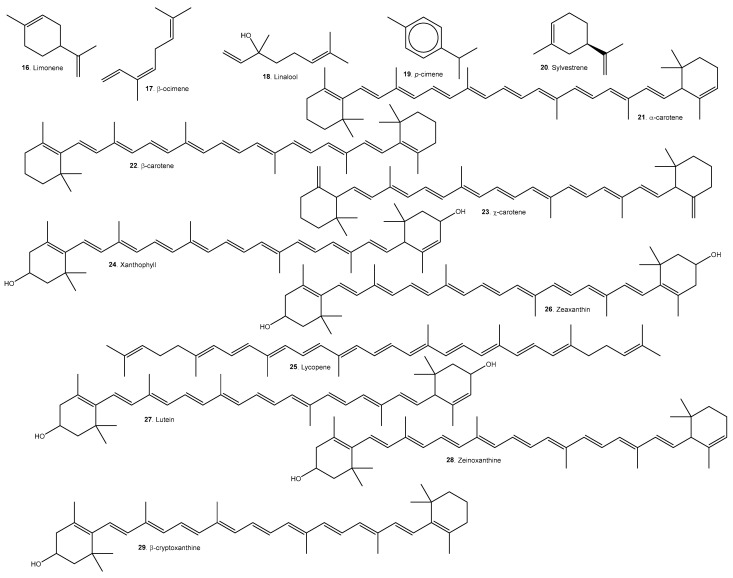
Terpenoids and carotenoids present in the selected species.

**Figure 9:**
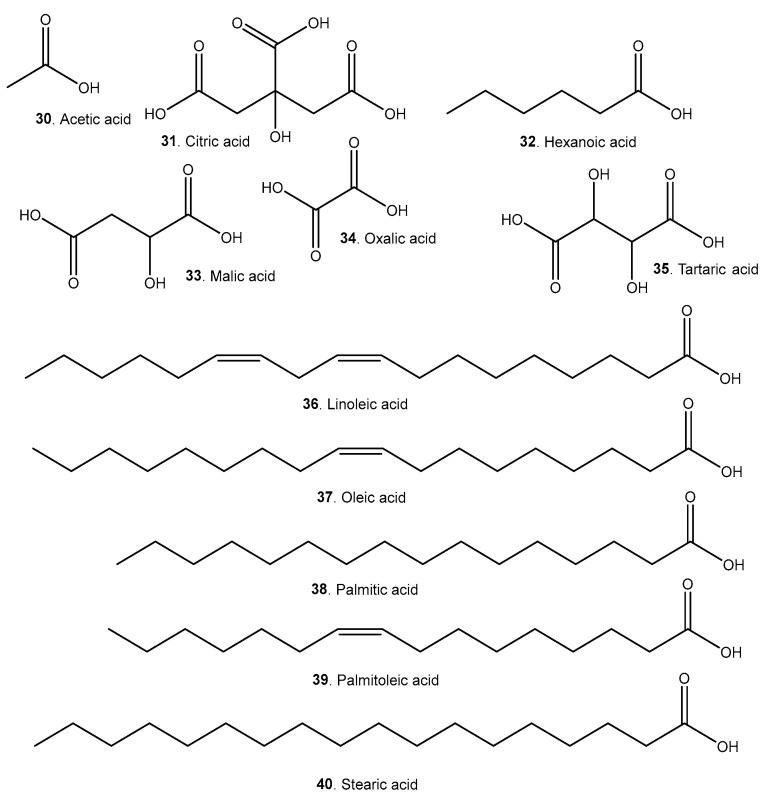
Simple carboxylic acids and fatty acids present in the studied fruits.

**Figure 10:**
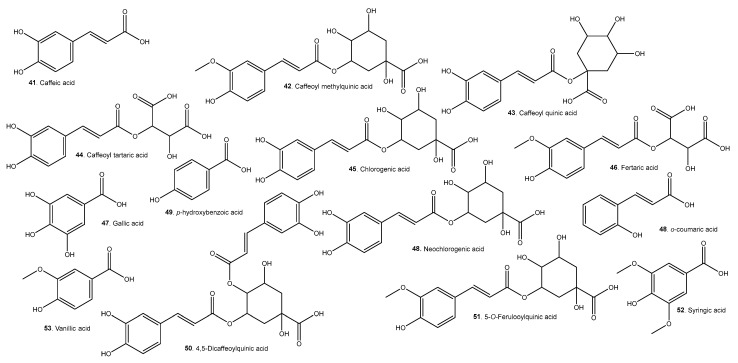
Phenolic acids present in the studied fruits.

**Figure 11:**
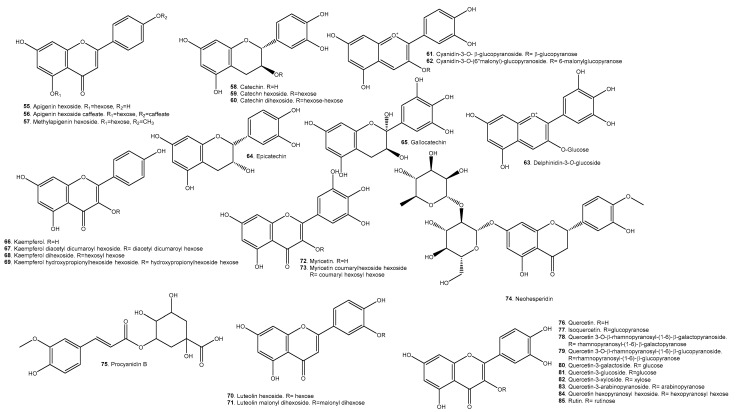
Flavonoids present in the studied fruits.

**Figure 12:**
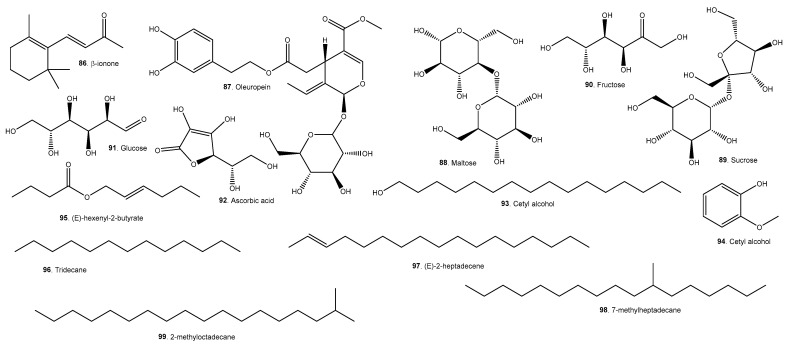
Other compounds present in the studied fruits.

A list of representative compounds in the studied species can be found in Table 6. They are divided into esters, alcohols (Figure 7); terpenoids and carotenoids (Figure 8); carboxylic acids (Figure 9); phenolic acids (Figure 10); flavonoids (Figure 11); and other compounds (Figure 12).

*A. Patinoi*, *E. stipitata*, and *S. sessiliflorum* contain a larger variety of esters. *A. patinoi* and *B. gasipaes* appear to have more alcohols (Figure 7). *B. gasipaes* and to a lesser extent *E. stipitata* contain more carotenoids than the other species (Figure 8). *A. patinoi* is rich in short-chain acids, and *B. gasipaes* in fatty acids (Figure 9) but shows no phenolic acids, which are found in variety in *A. patinoi*, *E. stipitata*, and *P. cecropiifolia* (Figure 10). *P. cecropiifolia* shows anthocyanins, attested by the color of the fruit, which have been studied for their anticancer activity [81]. *E. stipitata* appears to have the most varied flavonoid content among the studied fruits (Figure 11). Other compounds such as simple sugars, hydrocarbons, and higher alcohols are shown in Figure 12.

Esters are recognized as flavor and aroma compounds, and also exhibit antibacterial and anti-inflammatory activity. Methyl salicylate derivatives exhibit anti-inflammatory and analgesic activity [113].

Terpenoids exhibit a range of biological activity [114]. Particularly, *p*-cymene shows several properties related to ameliorating the impact of the Western dietary pattern: anti-inflammatory, antidiabetic, and antitumor among them [115].

Carotenoids have ample biological activity as antioxidants. Their intake, especially of lycopene, can reduce the risk of several chronic diseases linked to the Western dietary pattern: cardiovascular and neurological disorders, type 2 diabetes, and different types of cancer [96,116].

Heart-healthy fatty acids such as (**37**), especially in combination with polyphenols, can prevent and improve cardiovascular disease [117]. Other unsaturated fatty acids found in *B. gasipaes* can also ameliorate the effects of MetS [118,119].

Flavonoids are the most studied phenolic compounds, with a myriad of applications as antioxidants, anti-inflammatory, protective, and useful against several expressions of MetS [37,107,120].

Ascorbic acid is a powerful antioxidant and micronutrient. Oleuropein is a biologically active antioxidant, with antiproliferative activity and with testosterone-increasing activity that may partially underlie the fame of *Borojó* as an aphrodisiac [121,122,123].

Some current pharmacological uses of antioxidant compounds found in the studied fruits are listed in Table 7.

## 6. Patents

A patent search in Patentscope provides the following results.

*A. patinoi* shows 43 patents, most of which are for cosmetics and skin care products (22), followed by herbal remedies (11) and nutraceuticals (5). Some of the existing patents target conditions attributable to MetS, such as blood sugar, blood triglycerides, and diabetes [134]. Cosmetics patents include creams, supplements, and toothpaste [135]. Most of the patents (37) have been requested from China.

*B. gasipaes* shows nine patents, seven of which are unique. Four of the patents deal with food products, and one each with antibacterial and fungicides, packaging, and a solvent. The patents for these species use residues to create value, which is in line with SDG 12.

*E. stipitata* shows five patents, four of which are unique: food packaging [136], two low-calorie sweeteners, and packaging (same patent as for *B. gasipaes*).

*P.* cecropiifolia shows no patents.

*S. sessiliflorum* shows six patents: a hot sauce [137], food packaging [136], three patents for drought-resistant plants, and one for disease-resistant plants.

None of the existing patents make explicit use of the bioactive compounds present in the studied species. The numerous *A. patinoi* patents as herbal remedies may have more to do with the purported status of the species as a superfood and aphrodisiac, already identified with marketing rather than with science, than with the actual phytochemicals and scientifically demonstrated biological activity of the species.

## 7. Trends and Future Directions

The latest published research on the studied species shows trends associated with their commercial value. The latest studies on *A. patinoi* are concerned with the integration of the fruit into the food industry through the development of food products such as pastries, drinks, and confections, and with the aphrodisiac reputation of the fruit. *A. patinoi* latest studies are concerned with the integration of the fruit into the food industry through the development of food products such as pastries, drinks, and confections [20,138,139]. *B. gasipaes*, the most studied of the five species, is the subject of research on resistance to climate change, biofilm production, use of its starch in the production of aerogel, and other sustainable applications of a resource with waste that can be turned into new products [19,140,141]. *E. stipitata* garners interest for its phenolic compounds and essential oil, with research in microparticles, the insecticidal activity of its oil, and cultivation of the species [142,143,144]. *P. cecropiifolia* has been recently studied as a “superfood”, as a source of functional compounds, as an Acetylcholinesterase inhibitor, and as a crop susceptible of cultivation [82]. *S. sessiliflorum* is currently studied for its functional activity (hypolipemiant, antidiabetic, and antibacterial) and its phytochemical content [145,146,147].

*E. stipitata* is seeing increased interest in the last five years, as well as the study of carotenoids and antidiabetic and cholesterol-lowering properties in *B. gasipaes* [43].

**Figure 13:**
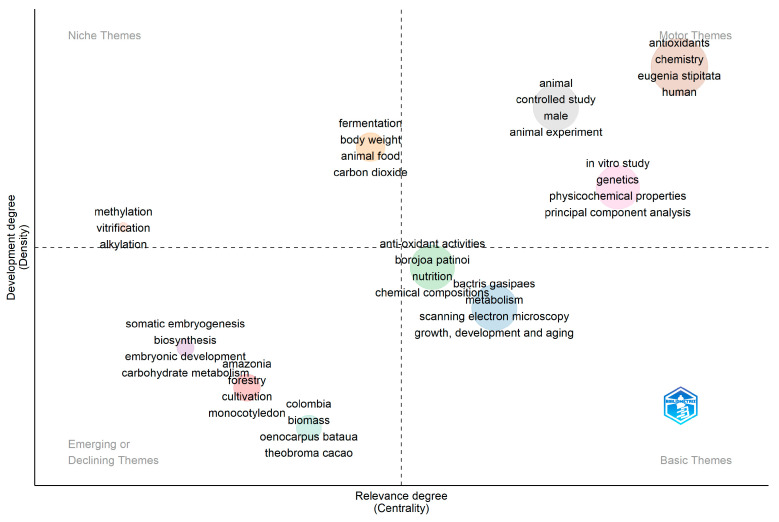
Thematic map of the study. Bibliometrix 4.0.

A thematic map shown in Figure 13 plots the published research on two axes: relevance degree and development degree, and shows four quadrants: Motor themes—well developed and important themes for the structuring of a research field, Niche themes—highly developed and specialized themes, Disappearing or emerging themes, and Basic themes—foundational and transversal themes [148,149]. Among the motor themes for the studied species, we find nonhuman (i.e., animal model), *S. sessiliflorum*, antioxidants, *E. stipitata*, human studies, and *A. patinoi*. *B. gasipaes* appears as a central element in the thematic map. *P. cecropiifolia* is not represented. This suggests that the research landscape is evolving from in vitro to in vivo studies and that the interest in *E. stipitata* and *S. sessiliflorum* is a motor theme [150].

### 7.1. Practical Implications

This study suggests practical implications for industry and policy makers. Suggested interconnected initiatives are including the species in the food heritage conservation and promotion of healthy traditional diets as an alternative to the WPD, supporting the development and implementation of sustainable cultivation methods for the studied species at all scales, supporting the development of value-added root-to-branch products based on traditional and novel uses of the species, and harmonious and respectful integration of the species into modern food systems with provisions to adequately utilize the waste in the development of co-products [151,152,153].

### 7.2. Limitations

The selected sources do not include all the studies on the selected species but were chosen for their quality [154]. The authors hope that research from the global South will attain more reach in the near future [155,156,157].

## 8. Conclusions

The five fruits reviewed in this study hold potential against metabolic syndrome beyond the benefits imparted by increasing fruit consumption. Antioxidant phytochemicals impart functional properties to the reviewed fruits, and one of them, *A. patinoi,* is already marketed with the dubious claim of “superfood”, while *B. gasipaes* is being studied for its antidiabetic and hypolipemiant potential.

The research volume for the selected fruits is in accordance with their economic importance. This presents an opportunity to study and prospect less traded species, the potential of which may not have been discovered. The studied species are mainly studied as food, with engineering and biomedical research categories in a secondary position.

*A. patinoi* is the species with most patents —mostly from China— with emphasis on cosmetics and herbal remedies. The other species have much fewer patents and *P. cecropiifolia* has none.

The authors recommend a more developed validation of the ethnopharmacological uses of the studied fruits; for example, the aphrodisiac properties of *A. patinoi*, which are compelling but have not been tested in vivo. Also, insufficiently studied properties of *P. cecropiifolia* and its anthocyanin-rich extracts seem promising.

By validating the medicinal properties attributed to these fruits, this study encourages the consumption of locally available, nutrient-rich foods, thus promoting traditional Amazonian foods as an alternative to WEP. There are opportunities for further research and development, through the identification of bioactive compounds in these fruits that open avenues for further research and product development. This can lead to the creation of new food products, supplements, or functional ingredients derived from these fruits. Understanding the bioactive potential of these underutilized fruits can also have economic and policy implications, potentially boosting the sustainable local agricultural sector and promoting cultural heritage through the preservation and utilization of traditional foods to enhance its economic and cultural significance.

## Figures and Tables

**Table 1:** Most common antioxidants from plant sources and their action mechanisms.

Antioxidant	Family/Examples	Mechanism	Chemical Feature	References
Phenolic acids	Salicylic, gentisic, *p*-hydroxybenzoic, protocatechuic, vanillic, syringic, gallic, *p*-coumaric, ferulic, caffeic and synapic acids	Hydrogen donors or transfer single electrons	Carboxylic acids with a hydroxy-substituted benzene ring within their structure	[15,33,36]
Flavonoids	Flavone (apigenin) Flavanol (epicatechin) Flavanone (naringenin) Flavanonol (taxifolin) Flavanol (quercetin) Isoflavone (genistein) Anthocyanidin (cyanidin)	Suppression of the formation of ROS or scavenging of ROS	Three rings of carbon atoms (C6-C3-C6) with additions of functional groups	[14,15,33,37]
Carotenoids	Lycopene, lutein, zeaxanthin, β-carotene	Scavenge singlet oxygen O^2^ and peroxyl-radicals by physical quenching	Carbon chain of conjugated carbon bonds	[15,33,36]
Tannins	Gallic acid, tannic acid, epigallocatechin	Donate hydrogen and electrons, chelate iron, and inhibit the activity of cyclooxygenase	Polymerization of phenylpropanoid compounds	[15,33,36]
Stilbenes	Piceid, resveratrol, piceatannol, and pterostilbene	Electron donor or enzyme activation	1,2-diphenylethylene structure	[15,33,36]
Ascorbic acid	-	Electron donor, scavenging free radicals	Vicinal OH groups	[15,33,36]
Vitamin E	Tocopherols tocotrienols	Donate hydrogen to lipid free radicals	Phenolic and a heterocyclic ring, conjugated with a phytyl chain	[15,33,36]

ROS: Reactive oxygen species; C: Carbon; O^2^: Oxygen; OH: Hydroxyl group.

**Table 2:** Literature review scheme. SPAR-4-SLR protocol.

Stage	Substage	
1 Assembling	1a Identification	Domain: health sciences, food science, ethnopharmacology, phytochemistry, phytomedicine Research question: What is the current knowledge about the potential against MetS of five underutilized Ecuadorian Amazon fruits *Borojó*, *chonta*, *arazá*, tree grape, and *cocona*? Source type: → Included: research articles, reviews, and book chapters → Excluded: preprints, proceedings Source quality: Crossref, Scopus databases
	1b Acquisition	Search mechanism and material acquisition: Dimensions, Scopus. Abstract and keyword queries. Search period: 2011–2024 Search keywords: (Borojoa OR Alibertia) AND patinoi, Bactris AND gasipaes, Eugenia AND stipitata, Pourouma AND cecropiifolia, Solanum AND sessiliflorum Total number of articles returned from the search: 554
2 Arranging	2a Organization	Organizing codes: ANZSRC, SDG.
	2b Purification	Article type excluded (*n* = 444): duplicates, remove predatory titles, remove non-empirical, non-review articles. Remove articles not directly related to the topic (cosmetics, plant genetics, pest control, etc.). Article type included (*n* = 110): Triangulation with previous reviews to ensure seminal articles are included [19,20,40,41,42,43].
3 Assessing	3a Evaluation	Analysis method: Content—descriptive Agenda proposal method: Future research directions, identification of existing gaps, practical applications.
	3c Reporting	Reporting conventions: Discussion and summaries in the form of tables and figures. Limitations: Discussed Sources of support: Acknowledged

**Table 3:** Research volume for the studied species.

Species	Documents	Top 5 Research Categories	Top SDGs
*A. patinoi* (Ap)	8	30 3006 31 40 32	15 2 7 12 13
*B. gasipaes* (Bg)	57	30 31 3006 3004 3108	2 14 15
*E. stipitata* (Es)	31	30 3006 3008 31 3004	15 3
*P. cecropiifolia* (Pc)	6	30 31 3004 34 3006	2 15
*S. sessiliflorum* (Ss)	20	30 3008 31 3004 3006	14

Note: Research categories are: 30, Agricultural, Veterinary, and Food Sciences. 31, Biological Sciences. 32, Biomedical and Clinical Sciences. 40, Engineering. 3004, Crop and Pasture Production. 3006 Food Sciences. 3008, Horticultural Production. 3108, Plant biology [45]. Sustainable Development Goals are: SDG 2: Zero Hunger. SDG 3: Good Health and Well-Being. SDG 7: Affordable and Clean Energy. SDG 12: Responsible Consumption and Production. SDG 13: Climate Action. SDG 14: Life Below Water. SDG 15: Life on Land [49]. Sum of document by species is larger than total documents because some studies include more than one species.

**Table 4:** Nutritional properties of the selected species.

Species	Energy (kcal/100 g)	Carbohydrate (g/100 g)	Protein (g/100 g)	Fat (g/100 g)	Vitamins (/100 g)	Minerals (mg/100 g)	Ref.
Ap	127.4	28.9	1.18	0.05	A: 253 UI C: 12.4 mg	Ca: 18.1 P: 18.6 Fe: 18.1	[20,70]
Bg	185–196	37.6 -41.7	2.6–3.3	4.3–4.6	A: 1117–3000 UI B9: 34 mg	K: 196 Mg: 20 Ca: 14–26	[19,71,72]
Es	15.6	3.6	0.71	0.3	A: 150 UI C: 36.8 mg	K: 827 Fe: 3.74 Mg: 76 Ca: 126 Cu: 1.12	[71,73,74,75]
Pc	36.6	15.5	0.3	0.4	B3: 1.2 mg C: 6 mg	K: 127 P: 32	[74,76]
Ss	33	5.7	0.6	1.4	A: 92 µg C: 14 mg B3: 2.5 mg	K: 1710 P: 1 Ca: 121	[77,78,79]

Note: All values are fresh weight.

**Table 6:** Representative phytochemicals of the studied fruits.

N°	Compound	Ap	Bg	Es	Pc	Ss
Esters						
**1.**	Benzyl acetate	X				
**2.**	Ethyl decanoate					X
**3.**	Ethyl hexanoate			X		
**4.**	Ethyl octanoate	X		X		X
**5.**	Ethyl propionate					X
**6.**	Ethyl-2-methylbutanoate			X		
**7.**	Hexyl acetate			X		
**8.**	Hexyl butyrate					X
**9.**	Methyl salicylate		X		X	X
Alcohols						
**10.**	1-hexanol	X	X	X		
**11.**	2-heptanol	X				
**12.**	2-nonanol	X				
**13.**	2-phenylethanol		X			X
**14.**	2,3-butanediol		X			
**15.**	1-nonanol		X			
Terpenoids						
**16.**	Limonene	X				
**17.**	β-ocimene			X		
**18.**	Linalool				X	X
**19.**	*p*-cymene					X
**20.**	Sylvestrene					X
Carotenoids						
**21.**	α-carotene			X		
**22.**	β-carotene		X	X		X
**23.**	γ-carotene		X			
**24.**	Xanthophyll		X			
**25.**	Lycopene		X			X
**26.**	Zeaxanthin			X		
**27.**	Lutein			X		
**28.**	Zeinoxanthin			X		
**29.**	β-cryptoxanthin			X		
Carboxylic acids						
**30.**	Acetic acid	X				
**31.**	Citric acid	X		X		X
**32.**	Hexanoic acid	X				
**33.**	Malic acid	X				
**34.**	Oxalic acid	X				
**35.**	Tartaric acid	X				
Fatty acids						
**36.**	Linoleic acid		X		X	
**37.**	Oleic acid		X			
**38.**	Palmitic acid		X		X	
**39.**	Palmitoleic acid		X			
**40.**	Stearic acid		X			
Phenolic acids						
**41.**	Caffeic acid	X				X
**42.**	Caffeoyl methylquinic acid			X		
**43.**	Caffeoyl quinic acid			X		
**44.**	Caffeoyl tartaric acid			X		
**45.**	Chlorogenic acid (not specified)	X			X	X
**46.**	Fertaric acid			X		
**47.**	Gallic acid			X		X
**48.**	Neochlorogenic acid				X	
**49.**	*o*-coumaric acid	X				
**50.**	*p*-hydroxybenzoic acid	X				
**51.**	Quinic acid, 4,5-*O*-dicaffeoyl				X	
**52.**	Quinic acid, 5-*O*-feruloyl				X	
**53.**	Syringic acid	X				
**54.**	Vanillic acid	X		X		
Flavonoids						
**55.**	Apigenin hexoside			X		
**56.**	Methylapigenin hexoside			X		
**57.**	Apigenin hexoside caffeate			X		
**58.**	Catechin	X			X	X
**59.**	Catechin hexoside			X		
**60.**	Catechin dihexoside			X		
**61.**	Cyanidin-3-*O*-β-glucopyranoside				X	
**62.**	Cyanidin-3-*O*-(6″malonyl)-glucopyranoside				X	
**63.**	Delphinidin-3-*O*-β-glucoside				X	
**64.**	Epicatechin				X	
**65.**	Gallocatechin			X		
**66.**	Kaempferol			X		
**67.**	Kaempferol diacetyl dicoumaroyl hexoside			X		
**68.**	Kaempferol dihexoside			X		
**69.**	Kaempferol hydroxypropionylhexoside hexoside			X		
**70.**	Luteolin hexoside			X		
**71.**	Luteolin malonyl dihexoside			X		
**72.**	Myricetin			X		
**73.**	Myricetin coumarylhexoside hexoside			X		
**74.**	Neohesperidin	X				
**75.**	Procyanidin B					X
**76.**	Quercetin			X		X
**77.**	Isoquercetin	X				
**78.**	Quercetin 3-*O*-α-rhamnopyranosyl-(1-6)-β-galactopyranoside				X	
**79.**	Quercetin 3-*O*-α-rhamnopyranosyl-(1-6)-β-glucopyranoside				X	
**80.**	Quercetin-3-galactoside				X	
**81.**	Quercetin-3-glucoside				X	
**82.**	Quercetin-3-xyloside				X	
**83.**	Quercetin-3-arabinopyranoside				X	
**84.**	Quercetin hexopyranosyl hexoside			X		
**85.**	Rutin	X			X	X
Other						
**86.**	β-ionone		X			
**87.**	Oleuropein	X				
**88.**	Maltose	X				
**89.**	Sucrose	X				X
**90.**	Fructose	X				X
**91.**	Glucose	X				X
**92.**	Ascorbic acid	X				X
**93.**	Cetyl alcohol				X	
**94.**	Guaiacol					X
**95.**	(E)-hexenyl-2-butyrate					X
**96.**	Tridecane					X
**97.**	(E)-2-heptadecene					X
**98.**	7-methylheptadecane					X
**99.**	2-methyloctadecane					X
**100.**	2-methyleicosane					X
**101.**	Pectin					X
**102.**	Nonanal		X			X

Note: Ap, *A. patinoi*. Bg, *B. gasipaes*. Es, *E. stipitata*. Pc, *P. cecropiifolia*. Ss, *S. sessiliflorum*. Ap sources [53,109]. Bg sources [68,69]. Es sources [69]. Pc sources [76,81,101]. Ss sources [78,110,111,112].

**Table 7:** Current uses of select antioxidant compounds found in the studied fruits against MetS-related disorders.

Antioxidant	Action	Targeted Conditions	Present in	Ref.
Chlorogenic acid	Lowering of glycemic impact of foods, lowering of background glucose level	Type 2 diabetes in a commercial product: Emulin™	Ap, Pc, Ss	[107]
Rutin	Nephroprotective	MetS-related kidney damage	Ap, Pc, Ss	[108]
Quercetin	Ameliorates MetS-related changes	Cardiovascular, hepatic, metabolic	Es, Ss	[124]
Myricetin	Antioxidant, anti-inflammatory, anticancer	Atherosclerosis, hypertension, ischemic heart disease	Es	[125,126]
Kaempferol	Anticoagulant, anti-platelet, antioxidant	Cardiovascular diseases associated with hyperactivation of platelets	Es	[127]
Apigenin	Antioxidant, anti-inflammatory anti-hypercholesterolemia	Atherosclerosis	Es	[128]
Gallocatechin	Vasorelaxation, antioxidant, anti-inflammatory	Hypertension	Es	[129]
Luteolin	Hypolipidemic, antioxidant, anti-atherosclerotic, hypotensive, diuretic	Ischemic cardiac disease, hyperlipidemia	Es	[130,131]
Cyanidins	Anti-inflammatory, antioxidant	Ischemic heart disease, hypertension	Pc	[132]
Anthocyanins, flavan-3-ols, flavanols, chlorogenic acids	Reduction in the expression of OC marker genes (calcitonin receptor, cathepsin K and RANK)	Osteoclastogenesis	Ap, Es, Pc, Ss	[133]

## Data Availability

No new data were created or analyzed in this study. Data sharing is not applicable to this article.
